# Exploring Relationships between Canopy Architecture, Light Distribution, and Photosynthesis in Contrasting Rice Genotypes Using 3D Canopy Reconstruction

**DOI:** 10.3389/fpls.2017.00734

**Published:** 2017-05-17

**Authors:** Alexandra J. Burgess, Renata Retkute, Tiara Herman, Erik H. Murchie

**Affiliations:** ^1^Division of Plant and Crop Sciences, School of Biosciences, University of NottinghamLoughborough, UK; ^2^Crops For the FutureSemenyih, Malaysia; ^3^School of Life Sciences, The University of WarwickCoventry, UK; ^4^School of Biosciences, University of Nottingham Malaysia CampusSemenyih, Malaysia

**Keywords:** 3D reconstruction, canopy architecture, crop productivity, light environment, MAGIC population, photosynthesis, rice (*Oryza* spp.)

## Abstract

The arrangement of leaf material is critical in determining the light environment, and subsequently the photosynthetic productivity of complex crop canopies. However, links between specific canopy architectural traits and photosynthetic productivity across a wide genetic background are poorly understood for field grown crops. The architecture of five genetically diverse rice varieties—four parental founders of a multi-parent advanced generation intercross (MAGIC) population plus a high yielding Philippine variety (IR64)—was captured at two different growth stages using a method for digital plant reconstruction based on stereocameras. Ray tracing was employed to explore the effects of canopy architecture on the resulting light environment in high-resolution, whilst gas exchange measurements were combined with an empirical model of photosynthesis to calculate an estimated carbon gain and total light interception. To further test the impact of different dynamic light patterns on photosynthetic properties, an empirical model of photosynthetic acclimation was employed to predict the optimal light-saturated photosynthesis rate (*P*_*max*_) throughout canopy depth, hypothesizing that light is the sole determinant of productivity in these conditions. First, we show that a plant type with steeper leaf angles allows more efficient penetration of light into lower canopy layers and this, in turn, leads to a greater photosynthetic potential. Second the predicted optimal *P*_*max*_ responds in a manner that is consistent with fractional interception and leaf area index across this germplasm. However, measured *P*_*max*_, especially in lower layers, was consistently higher than the optimal *P*_*max*_ indicating factors other than light determine photosynthesis profiles. Lastly, varieties with more upright architecture exhibit higher maximum quantum yield of photosynthesis indicating a canopy-level impact on photosynthetic efficiency.

## Introduction

The rate of photosynthesis of a given stand of crops is dependent on a multitude of factors including weather, temperature, leaf age, and plant development. Photosynthesis, in turn, is closely linked to potential yield (Murchie et al., 2009; Zhu et al., 2010). However, the complex arrangement of overlapping leaves of different ages and in different states of photosynthesis means that assessing canopy level photosynthesis from individual leaf activity is difficult and time consuming. For an accurate prediction of canopy photosynthesis from leaf measurements, it is necessary to have data on multiple leaf characteristics including physical orientation, positioning and physiological characteristics, such as photosynthetic acclimation and nutrient status (Burgess et al., 2015, 2016). However, predicted productivity tends to be higher than that measured in the field (Zhu et al., 2010). The cause of this disparity is unclear, but may arise from suboptimal photosynthetic responses to dynamic environmental changes partly caused by architectural traits (Zhu et al., 2010; Burgess et al., 2015).

In the absence of methods for whole canopy measurements, such as in Song et al. (2016), predictions require knowledge of the architectural characteristics and its effect on canopy light distribution. Photosynthetic rate is highly sensitive to light intensity, and, in turn, the light intensity within crop canopies has high spatio-temporal variability, and is dependent upon features such as leaf angle, size and shape, leaf number, and the arrangement of this material in three-dimensional space. These findings have led to the concept of an “idealized plant type” or “ideotype.” For example, the International Rice Research Institute (IRRI) proposed that upright leaves, large panicles and fewer tillers would represent the ideal structure for rice (Dingkuhn et al., 1991; Virk et al., 2004). Erect leaf morphology is a characteristic that repeatedly arises within the concept of an ideotype. This is due to the increased light penetration to deeper canopy layers leading to uniformity of light within the canopy setting and maximal net photosynthesis (Clendon and Millen, 1979; Hodanova, 1979; Turitzin and Drake, 1981; Setter et al., 1995; Normile, 1999). Within dense canopies, steeper leaf angles potentially lead to an improvement in whole day carbon gain by enhancing light absorption at low solar angles (Falster and Westoby, 2003). Erect leaf stature is also associated with reduced susceptibility to photoinhibition and reduced risk of overheating (King, 1997; Murchie et al., 1999; Werner et al., 2001; Falster and Westoby, 2003; Burgess et al., 2015). As such, the erect ideotype is predicted to be most effective in low latitudes but it has also been found to be productive in high latitudes (Reynolds and Pfeiffer, 2000; Peng et al., 2008 and references within). However, despite this, there is still variation in crop morphology and the erect ideotype is not widespread in many species. As such, there may still be potential for yield improvement by alteration of canopy architectural characteristics (Reynolds et al., 2000; Khush, 2005; Khan et al., 2015; Rötter et al., 2015).

There is currently no method for producing accurate high-resolution 3D architectural reconstructions of entire field grown crop canopies via imaging techniques for modeling purposes. This is largely due to problems of occlusion at high leaf densities i.e., of being unable to produce images of leaves deep within the canopy using the most common optical techniques. Being able to do so would be highly advantageous for testing hypothesis about canopy structure within fundamental or applied research. However, advances in hardware and image processing have led to new methods for capturing and evaluating plant architecture. These methods have been used for numerous purposes including both plants grown in pots and those grown under field conditions (e.g., Falster and Westoby, 2003; Godin and Sinoquet, 2005; Watanabe et al., 2005; Quan et al., 2006; Sinoquet et al., 2007; Zheng et al., 2008; Burgess et al., 2015). Whilst previous studies have attempted to look at the relationship between canopy architecture and the light environment (e.g., Zheng et al., 2008; Song et al., 2013), these have been restricted due to the relatively inaccurate manual reconstruction and modeling techniques used and the limited genetic variation and architectural types studied. Architectural traits are inherently linked to the resulting light environment and since photosynthetic rate is strongly light-dependent it therefore follows that photosynthetic rate will be dependent upon architecture.

To overcome the limitations of previous studies we used a new approach for high resolution 3D reconstruction of crop plants (Pound et al., 2014; Burgess et al., 2015) to investigate fundamental structure-function canopy properties. This is not a high throughput technique but rather uses individual plants extracted from field grown plots to generate highly accurate representations that can then be used to populate a canopy *in silico* for ray-tracing and photosynthesis modeling. The parental lines used for the creation of multi-parent advanced generation inter-cross (MAGIC) populations in rice (Bandillo et al., 2013) were selected for analysis within this study. These lines have a well-researched genetic background and contain desirable traits for yield, grain quality, and biotic and abiotic stress resistance (more details on each line are given in Supplementary Table S1). Furthermore, the contrasting origin of each line means that they are cultivated in diverse habitats with different stressors and constraints. The initial phase of this study involved a preliminary small-scale screening experiment to assess differences in terms of architectural and physiological features for 15 of the lines (referred to here as M1–M15 in Supplementary Table S1). Four of these lines, Shan-Huang Zhan-2 (SHZ-2), IR4630-22-2-5-1-3, WAB 56–125, and Inia Tacuari (referred to here as M2, M4, M11, and M13, respectively), plus the Philippine high-yielding variety IR64 were chosen for an in depth physiological study. These lines were chosen due to their differences in a number of features including leaf area index (LAI; leaf area per unit ground area), chlorophyll a:b ratios (a reliable indicator of shade acclimation state, reflecting the proportion of chlorophyll in light harvesting complexes), chlorophyll content and physical appearance. The aims are to: (1) assess the method for image based reconstruction on genetically variable rice plants grown in simulated field environment (see materials and methods); (2) test the hypothesis that there are common links between canopy architecture and photosynthetic traits across genetically diverse rice cultivars (such as leaf angle, light distribution, and photosynthetic capacity) and; (3) test the hypothesis that canopy-induced dynamic light properties are associated with the acclimation status of leaves in genetically diverse cultivars. The latter uses a new empirical acclimation model which predicts the optimal *P*_*max*_ (if light were the sole determinant; Retkute et al., 2015). Acclimation is a process whereby leaves adjust their photosynthetic capacity, dark respiration and light compensation point according to long term changes in the light environment. However, the ability to acclimate optimally in fluctuating conditions has not been fully tested (Anderson et al., 1995; Murchie and Horton, 1997, 1998; Yano and Terashima, 2001; Walters, 2005; Athanasiou et al., 2010; Retkute et al., 2015).

## Materials and methods

### Plant material and growth

The preliminary screening used 15 of the possible 16 parental lines from a MAGIC rice population (Bandillo et al., 2013; details given in Supplementary Table S1 with results of the screening in Supplementary Table S2). Seeds were sown into module trays containing Levington Module compost [N (96 ppm), P (49 ppm), K (159 ppm)] mixed with 30% sand by volume in the FutureCrop Glasshouse facilities, University of Nottingham Sutton Bonington Campus (52°49′59″ N, 1°14′50″ W), UK on the 7th May 2015. The FutureCrop Glasshouse is a south—facing glasshouse designed and built by CambridgeHOK (Brough, UK) for the growth of crop stands within a controlled environment. It consists of a concrete tank 5 × 5 × 1.25 m positioned at ground level. The tank is filled entirely with a sandy loam soil, extracted from local fields, and sieved through a fine mesh. The seedlings were transplanted into microplots (containing 5 × 5 plants with 10 × 10 cm spacing between adjacent plants; 100 plants m^−2^) within soil beds 7 days after root establishment. For the preliminary screen, key measurements were made 55–60 days after transplanting (DAT), corresponding to a vegetative growth phase (Supplementary Table S2). Ten centimeters of spacing is consistent with rice field planting guidelines (www.irri.org). Following the preliminary screening, four lines; Shan-Huang Zhan-2 (SHZ-2), IR4630-22-2-5-1-3, WAB 56–125, and Inia Tacuari (referred to here as M2, M4, M11 and M13, respectively), were selected for the in depth study as well as the popular Philippine variety IR64, from IRRI. Selection was made largely on the basis of contrasting architecture including leaf area index (LAI; leaf area per unit ground area), chlorophyll a:b ratios and content plus physical appearance. This selection also represents rice from diverse origins (Supplementary Table S1) and genetic backgrounds (M2, M4 and IR64 of indica and M11 plus M13 of japonica). The seeds were sown into module trays on the 15th October 2015 and transplanted into replicate microplots of 6 × 6 plants (10 cm spacing as above) using a completely randomized design. Plots were arranged in a 3 × 4 design that minimized edge effects and plants on edge of plots were not used in this study. The glasshouse conditions were kept consistent for both the screening and the in depth study. Irrigation was supplied using drip irrigation for 15 min, twice daily. Sodium (Son T- Agro, Philips) lamps provided additional lighting whenever the photosynthetically active radiation (PAR) fell below 300 μmol m^−2^ s^−1^ and a 12 h photoperiod (07:00–19:00) was maintained using blackout blinds. A temperature of 28 ± 3°C and relative humidity (RH) of 50–60% was maintained throughout. Nutrient composition of plots was measured by sampling soil at leaf 3, during the vegetative growth stage. Consequently Yara Milla complex fertilizer (applied at rate equivalent to 50 kg ha^−1^ of N plus micronutrients) was applied to the plots, 80 days after transplanting (DAT).

### Physiological measurements: in depth study

In depth measurements were made at two different growth stages: 45 and 85 DAT, which correspond to an early (prior to full canopy development) and late (full canopy development prior to flowering) vegetative phase. Here, we refer to these stages as growth stage 1 (GS1) and growth stage 2 (GS2), terms used in this study only. Five replicate measurements of plant height per plot were taken weekly, from four DAT. Five replicate measurements per plot were taken for tiller numbers at each of the growth stages. Three replicate plants per line were taken for leaf width, leaf area, fresh, and dry weight measurements at each growth stage. Individual plant dry weight and area was analyzed by passing material through a leaf area meter (LI3000C, Licor, Nebraska) and drying in an oven at 80°C for 48 h or until no more weight loss was noted. Measured LAI (leaf area per unit ground area: m^2^ m^−2^) was calculated as the total area (leaf + stem) divided by the area of ground each plant covered (distance between rows × distance within rows) and averaged across the replicate plants. A Walz MiniPam fluorometer was used to measure dark-adapted values of Fv/Fm in the glasshouse at mid-day. Leaves were dark adapted using clips (DLC-08; Walz) for at least 20 min and Fo and Fm were measured by applying a saturating pulse (0.8 s, 6,000 μmol m^−2^ s^−1^). Five replicate measurements on different leaves were taken per plot. Chlorophyll a and b content and ratios were determined through chlorophyll assays corresponding to GS2. Frozen leaf samples of known area were ground in 80% acetone, centrifuged for 5 min at 1,600 g, and the absorbance (at 663.6 and 646.6 nm) of the supernatant was measured using a spectrophotometer according to the method of Porra et al. (1989).

### Imaging and ray tracing

3D analysis of a plant from each plot (i.e., three replicate plants per line which accounts for any within—genotype variability caused by environment) was made according to the protocol of Pound et al. (2014) based on stereo-imaging in the in-depth analysis (GS1 and GS2). Briefly, plants were removed carefully from the central part of the plots (with roots and soil). They were positioned on a calibration target and turntable. SLR cameras were placed at three positions and 45–60 images recorded as the plant was carefully rotated. Automated reconstruction of a 3-D point cloud and conversion of this to a 3D canopy representation made up of 2D flat leaves took place using existing software described in Pound et al. (2014). These reconstructions were duplicated and rotated to form a 3 × 3 canopy grid (with set 10 cm spacing between plants), with the same leaf area index (LAI) as the measured plants (see Table 1). The LAI of each reconstructed canopy was calculated as the area of mesh inside the ray tracing boundaries divided by the ground area. A forward ray-tracing algorithm, fastTracer (fastTracer version 3; PICB, Shanghai, China from Song et al., 2013), was used to calculate diurnal change in total light per unit leaf area throughout the canopies. Latitude was set at 14.2 (for the Philippines), atmospheric transmittance 0.5, light scattering 7.5%, light transmittance 7.5%, days 344 (GS1 10th December), and 21 (GS2 21st January). The diurnal course of light intensities over a whole canopy was recorded at 1 min intervals. The aim was to study the effect of canopy architecture on the resultant light environment and the impact on whole canopy photosynthesis thus the same parameters for ray tracing were used for each of the canopies, despite the diverse origin of each of the lines (see Supplementary Table S1).

**Table 1:**
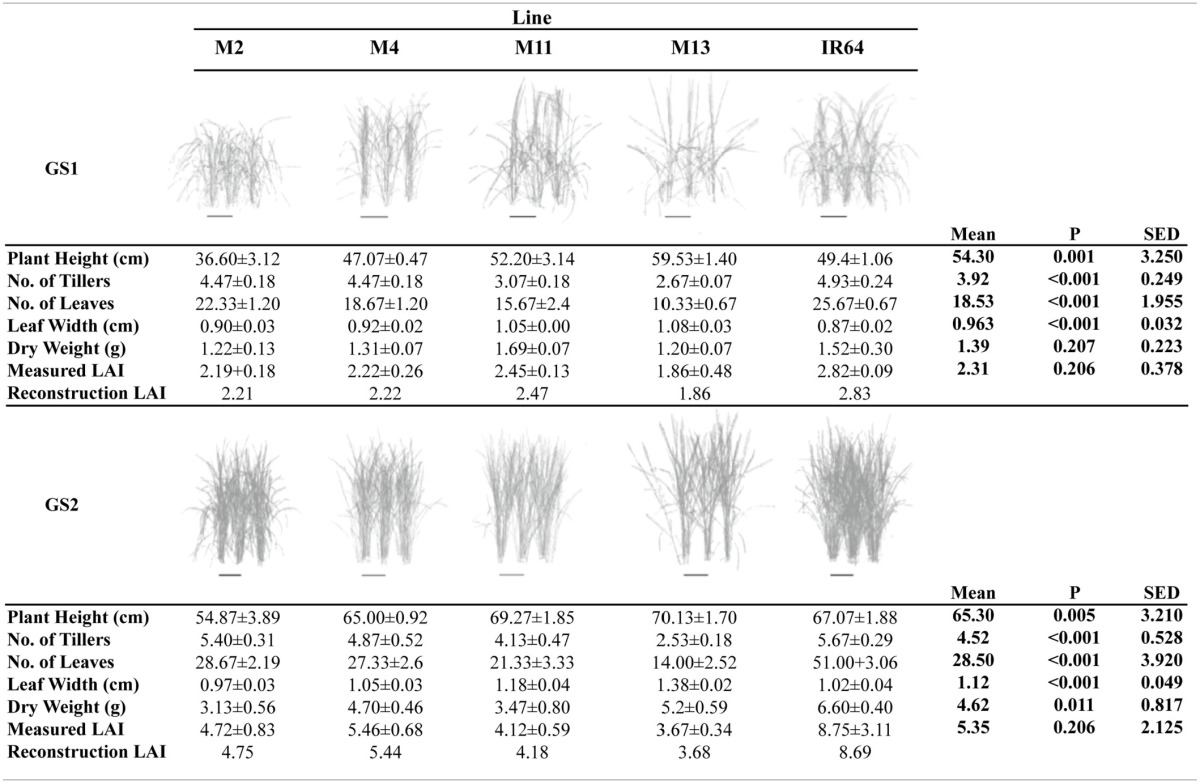
**Canopy reconstructions and description**.

*The means of three plots are shown with standard errors of the mean. P-values correspond to ANOVA. Plants were imaged and reconstructed as a single plant according to the protocol of Pound et al. (2014). These were then duplicated and rotated and arranged on a 3 × 3 canopy grid. Rotating the plants enabled a similar leaf area index (LAI) to be achieved. Measured LAI was calculated as the total area (leaf + stem), using a leaf area meter (LI3000C, Licor, Nebraska), divided by the area of ground each plant covered (distance between rows × distance within rows). The reconstructed LAI was calculated as mesh area inside the designated ray tracing boundaries (see Section Imaging and Ray Tracing). Following imaging and measurement of leaf area, dry weights were calculated. The resting plant height (the plant height in the natural position, i.e., taking into account leaf curling), number of tillers, number of leaves, and leaf width of five plants per plot was calculated. Growth stage 1 corresponds to 45 DAT and 2 at 85 DAT. M2, M4, M11, and M13 refer to Shan-Huang Zhan-2 (SHZ-2), IR4630-22-2-5-1-3, 157 WAB 56-125, and Inia Tacuari, respectively. Growth stage 1 corresponds to 45 DAT and 2 at 85 DAT*.

### Gas exchange

Photosynthesis-light response curves (LRC) and Photosynthesis vs. Ci (leaf internal CO_2_ concentration; ACi) curves were taken via infra-red gas exchange (IRGA). Leaves were not dark-adapted prior to measurements. LRCs were taken at GS1 and 2 whereas ACi curves were taken at GS1 only. Leaf gas exchange measurements (LRC and ACi) were taken with a LI-COR 6400XT infra-red gas-exchange analyser (LI-COR, Nebraska). The block temperature was maintained at 30°C using a flow rate of 500 ml min^−1^ and ambient humidity. For light response curves, light was provided by a combination of in-built red and blue LEDs. Illumination occurred over a series of 12 photosynthetically active radiation values (low to high), between 0 and 2,000 μmol m^−2^ s^−1^, with a minimum of 2 min and maximum of 3 min at each light level at two different canopy heights; top (center of flag leaf) and bottom (25% of full canopy height). Therefore, the positions were not affected by canopy height. Separate induction curves showed that this was sufficient to fully induce leaves. For the A-Ci curves; leaves were exposed to 1,500 μmol m^−2^ s^−1^ throughout. They were placed in the chamber at 400 p.p.m. CO_2_ for a maximum of 2 min and then CO_2_ was reduced stepwise to 50 p.p.m. CO_2_ was then increased to 1500 p.p.m. again in a stepwise manner. Two replicates were taken per layer per treatment plot for both sets of measurements apart from LRCs for GS2, which has five replicates overall for each of the five varieties.

### Statistical analysis

Analysis of variance (ANOVA) was carried out using GenStat for Windows, 17th Edition (VSN International Ltd.). Data was checked to see if it met the assumption of constant variance and normal distribution of residuals. A correlation matrix was used to investigate the relationships between different physiological traits.

### Modeling

All modeling was carried out using Mathematica (Wolfram).

Cumulative leaf area index (cLAI; leaf area per unit ground area as a function of depth) was calculated from each of the canopy reconstructions. cLAI was not measured in this study but previous work has validated this method using manual measurements of leaf area (Pound et al., 2014). Leaves are represented here as a series of small 2D triangles. For each depth (*d*; distance from the highest point of the canopy), all triangles with centers lying above *d* were found (Equation 1).

(1) $$d_{i}=\max_{j=1,2,3;1\leq i\leq n}z_{i}^{j}-\left(z_{i}^{1}+z_{i}^{2}+z_{i}^{3}\right)/3$$

The sum of the areas of these triangles was calculated and divided by the ground area. The cumulative LAI as a function of depth through the canopy was calculated using Equation (2).

(2) $$cLAI(d)=\;\frac{\sum_{i=1}^{n}I(d_{i}\leq d)S_{i}}{\left(\max_{1\leq i\leq n}\;x_{i}-\min_{1\leq i\leq n}\;x_{i})(\max_{1\leq i\leq n}y_{i}-\min_{1\leq i\leq n}\;y_{i}\right)}$$

where *I(A)* = *1* if condition *A* is satisfied and *S*_*i*_ is the area of a triangle *i*.

The light extinction coefficient of the canopy was calculated using the 3D structural data and the light distribution obtained from ray tracing. In order to calculate fractional interception (FI) within a canopy as a function of depth at time *t*, all triangles lying above depth, *d*, were identified (Equation 1). Their contribution to intercepted light was then calculated by multiplying PPFD received per unit surface area (ray tracing output) by the area of triangle. The light intercepted was summed for all triangles above the set *d*, and divided by light intercepted by ground area according to Equation (3).

(3) $$F(d,t)=\frac{\sum_{i\;=\;1}^{n}I\left(d_{i}\leq d\right)\;S_{i}L_{i}\left(t\right)}{L_{0}\left(t\right)\text{*}ground\;area}$$

where *L*_0_*(t)* is light received on a horizontal surface with a ground area (max_1 ≤ *i* ≤ *n*_*x*_*i*_−min_1 ≤ *i* ≤ *n*_ *x*_*i*_)(max_1 ≤ *i* ≤ *n*_ *y*_*i*_−min_1 ≤ *i* ≤ *n*_ *y*_*i*_), and *L*_*i*_(*t*) is light intercepted by a triangle *i*.

The light extinction coefficient, *k*, was calculated by fitting (by least squares) the function,

(4) $$f(x)\;=\;a\left(1-e^{-k\;x}\right)$$

to the set of points {*cLAI*(*d*), *F*(*d, t*)} calculated by varying depth from 0 to the height at total c*LAI* with step Δ*d* = 1 mm, *a* in Equation (4) is a fitted parameter.

The response of photosynthesis to light irradiance, *L*, was calculated using a non-rectangular hyperbola given by Equation (5):

(5) $$F_{NRH}\left(L,\phi ,\theta ,P_{max},\alpha \right)\;=\;\frac{\phi \;L+\left(1+\alpha \right)P_{max}-\sqrt{{\left(\phi L+\left(1+\alpha \right)P_{max}\right)}^{2}-4\theta \phi L\left(1+\alpha \right)P_{max}}\;}{2\theta }-\alpha P_{max}$$

Values for *P*_*max*_ were determined from leaf gas exchange measurements (see Section Gas Exchange). The value of α was obtained by fitting a line of best fit between all measured *P*_*max*_ and *Rd*-values. All other parameters (e.g., *P*_*max*_, Φ, and θ) were estimated from the light response curves for three canopy layers using the Mathematica command *FindFit*.

As each canopy was divided into two layers, and each triangle from the digital plant reconstruction was assigned to a particular layer, *m*, according to the triangle center (i.e. with triangle center between upper and lower limit of a layer depth). Carbon gain per unit canopy area was calculated as daily carbon assimilation over a whole canopy divided by the total surface area of the canopy according to Equation (6).

(6) $$C\;=\;\frac{\sum_{i=1}^{n}P_{i}}{\sum_{i=1}^{n}S_{i}}.$$

Total canopy light interception per unit leaf area over whole day was calculated according to Equation (7).

(7) $$TL_{LA}\;=\;\frac{\sum_{i=1}^{n}S_{i}\int_{6}^{18}L_{i}(t)dt}{\sum_{i=1}^{n}S_{i}}$$

where *S*_*i*_ is the area of triangle *i*.

An empirical model of acclimation was employed to predict the distribution of optimal *P*_*max*_-values throughout each of the canopies. Details of the model can be found in Retkute et al. (2015). The model can be used to predict the maximum photosynthetic capacity, $P_{max}^{opt}$, as the *P*_*max*_ that represents maximal carbon gain at a single point within the canopy, based on the light pattern that point has experienced (i.e., using the light pattern output from ray tracing). This was predicted across 250 canopy points, thus leading to distribution of $P_{max}^{opt}$ -values throughout each of the canopies. The canopy locations were chosen as a subset of triangles that were of similar size (i.e., area) and constitute a representative sample distribution throughout canopy depth.

Carbon gain, *C* (mol m^−2^) was calculated over a given time period (e.g., daily) *t* ε [6,18] (Equation 8).

(8) $$C(L(t),P_{max})\;=\;\int_{6}^{18}P(L(t),P_{max})dt$$

Experimental data indicates that the response of photosynthesis to a change in irradiance is not instantaneous and thus to incorporate this into the model Retkute et al. (2015) introduced a time-weighted average for light (Equation 9).

(9) $$L_{\tau }(\text{t})\;=\;\frac{1}{\tau }\;\int_{-\infty }^{t}L(t\prime )e^{-\frac{t-t\prime }{\tau }dt^{\prime }}$$

This effectively accounts for photosynthetic induction state, which is hard to quantify *in situ* as it varies according to the light history of the leaf. The more time recently spent in high light, the faster the induction response, thus the time-weighted average effectively acts as a “fading memory” of the recent light pattern using an exponentially decaying weight. If τ = 0 then a plant will able to instantaneously respond to a change in irradiance, whereas if τ > 0 the time-weighted average light pattern will relax over the timescale τ. Within this study, τ was fixed at 0.2 (unless otherwise stated) in agreement with previous studies and fit with past experimental data (Pearcy and Seemann, 1990; Retkute et al., 2015) and measurements of induction state in rice leaves. The time-weighted average only applies to the transition from low to high light; from high to low, response is instantaneous and does not use the weighted average (see Supplementary Figure S1). The model was parameterised using the convexity and dark respiration values taken from the fitted LRCs. A moving average of the *P*_*max*_ throughout canopy height was fitted using the Mathematica command *MovingAverage* to give an approximate relationship between canopy height and optimal *P*_*max*_ based on the light environment.

## Results

### Architectural features

#### Manual measurements

**Figure 1:**
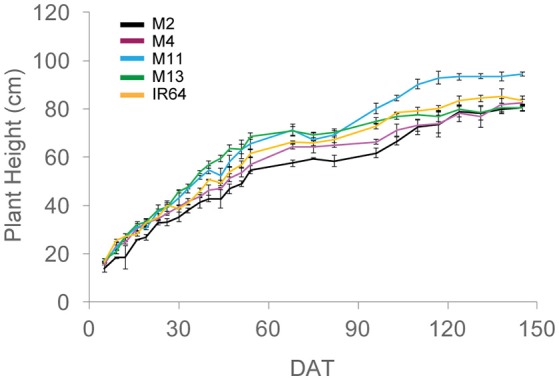
**Plant height over the course of the experiment, calculated as the average of five measurements per plot**. The means of three plots are shown with standard errors of the mean. M2, M4, M11, and M13 refer to Shan-Huang Zhan-2 (SHZ-2), IR4630-22-2-5-1- 3, 157 WAB 56-125, and Inia Tacuari, respectively.

A summary of the key architectural features is given in Table 1 (see Supplementary Table S2 for the initial screening experiment). Similarities can be seen between the key architectural features: the initial screening experiment and the in-depth study (Table 1 and Supplementary Table S2) however the variation seen between the lines was reduced in the second, in depth experiment. For the rest of the paper, only data from the in-depth study will be considered. Plant height varied between lines in both growth stages (*P* = *0.001* for GS1 and *P* = *0.005* for GS2), with M2 the shortest and M13 the tallest of the five lines. The change in plant height over the course of the experiment is given in Figure 1. One-hundred and fifty DAT is full maturity and just before harvest and the increase in height after 90 DAT likely corresponds to stem elongation. Height is a relevant architectural trait since upland cultivars can be taller than lowland, thought to be a trait associated with weed competition. Here, M11 has aerobic adaptation and M13 is NERICA i.e., derived partly from *Oryza glaberrima*. Since plant height infers greater stem and leaf sheath extension it may be an important trait in determining partitioning, available leaf area and productivity in a given environment. Leaf blade width differed between the lines at each growth stage (*P* < 0.001 GS1 and 2) with M11 and M13 exhibiting the widest leaf blades (Table 1). Leaf number and tiller number also differed significantly between the lines (*P* < 0.001 both growth stages) with M13 containing the fewest number of leaves and IR64 the greatest, however there was no significant difference in leaf area index (LAI) at either growth stage (Table 1). Dry matter was not significantly different between lines (Table 1) indicating that modeled photosynthesis was not a reliable predictor of biomass production in this case. This could be caused by a number of factors including lack of inclusion of partitioning of biomass to roots or measuring photosynthesis at a limited number of stages.

#### Modeled data

**Figure 2:**
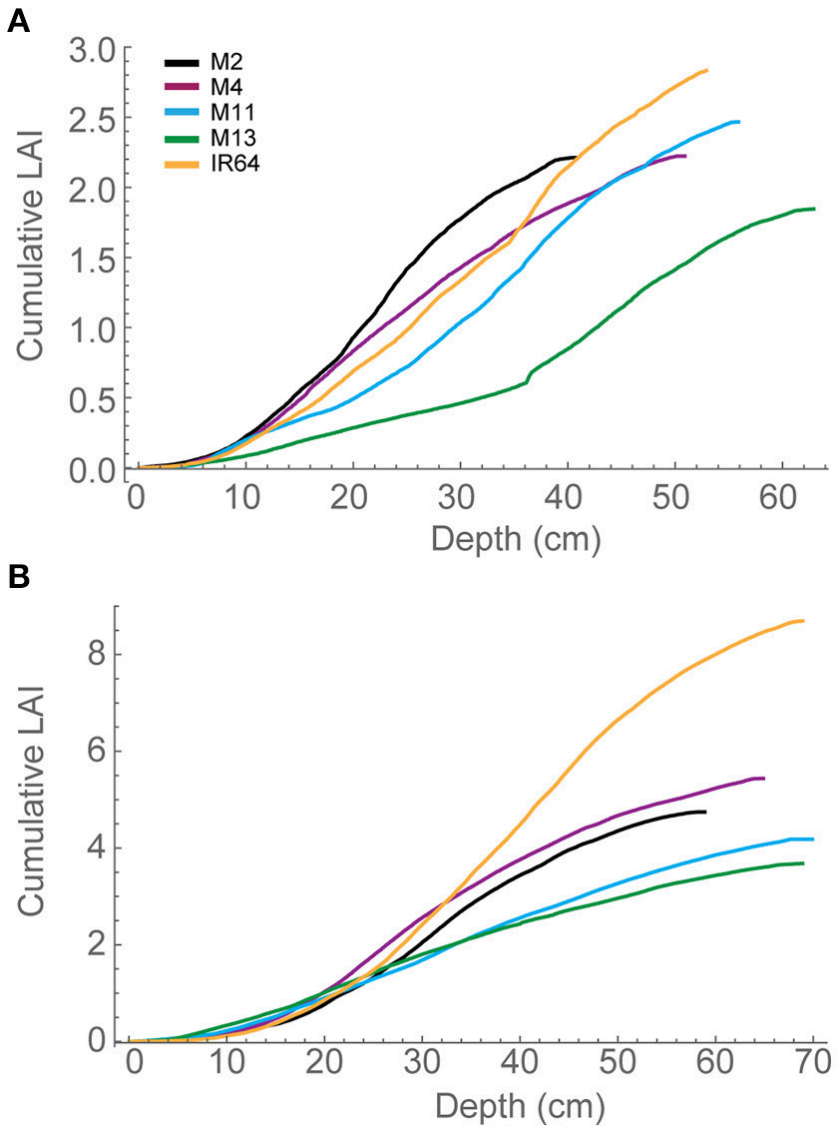
**Modeled cLAI, the area of leaf material (or mesh area) per unit ground as a function of depth through the canopy (i.e., distance from the top) at 12:00 h for (A)** GS1 and **(B)** GS2. M2, M4, M11, and M13 refer to Shan-Huang Zhan-2 (SHZ-2), IR4630-22-2-5-1- 3, 157 WAB 56-125, and Inia Tacuari, respectively.

Each plant within the *in silico* canopy was rotated around the vertical axis such that the LAI inside the ray tracing boundaries was consistent with measured data (Table 1; see Section Materials and Methods). Previous papers have validated the modeling using measured data of LAI and extinction coefficients (Burgess et al., 2015). Cumulative leaf area index (cLAI) was calculated through canopy depth (i.e., from top-down; see Section Modeling) for each of the canopies at each growth stage (see Figures 2A,B). A curve was deliberately not fitted because the reconstruction and modeling approach used within this study permits the actual relationship between LAI and depth in the canopy to be depicted, without the need for curve fitting. Generally, a sigmoidal response was seen for most genotypes with a more rapid accumulation of leaf area toward the center of the canopy. At GS1, M2, and M13 show the greatest difference among lines in terms of the position of accumulation of LAI according to depth (distance from the top of the canopy) with the latter accumulating more biomass in the bottom half of the canopy (Figure 2A). At GS2 (Figure 2B) this pattern is not pronounced with other lines showing a similar increase in cLAI up to ~20 cm depth. From here on, differences are shown with M11 and M13 exhibiting least accumulation of leaf material and IR64 exhibiting the greatest. This variation is consistent with total measured LAI-values, with IR64 exhibiting a much higher overall LAI compared to the other lines (Table 1), although according to ANOVA on the measured leaf area, this is not significant.

**Figure 3:**
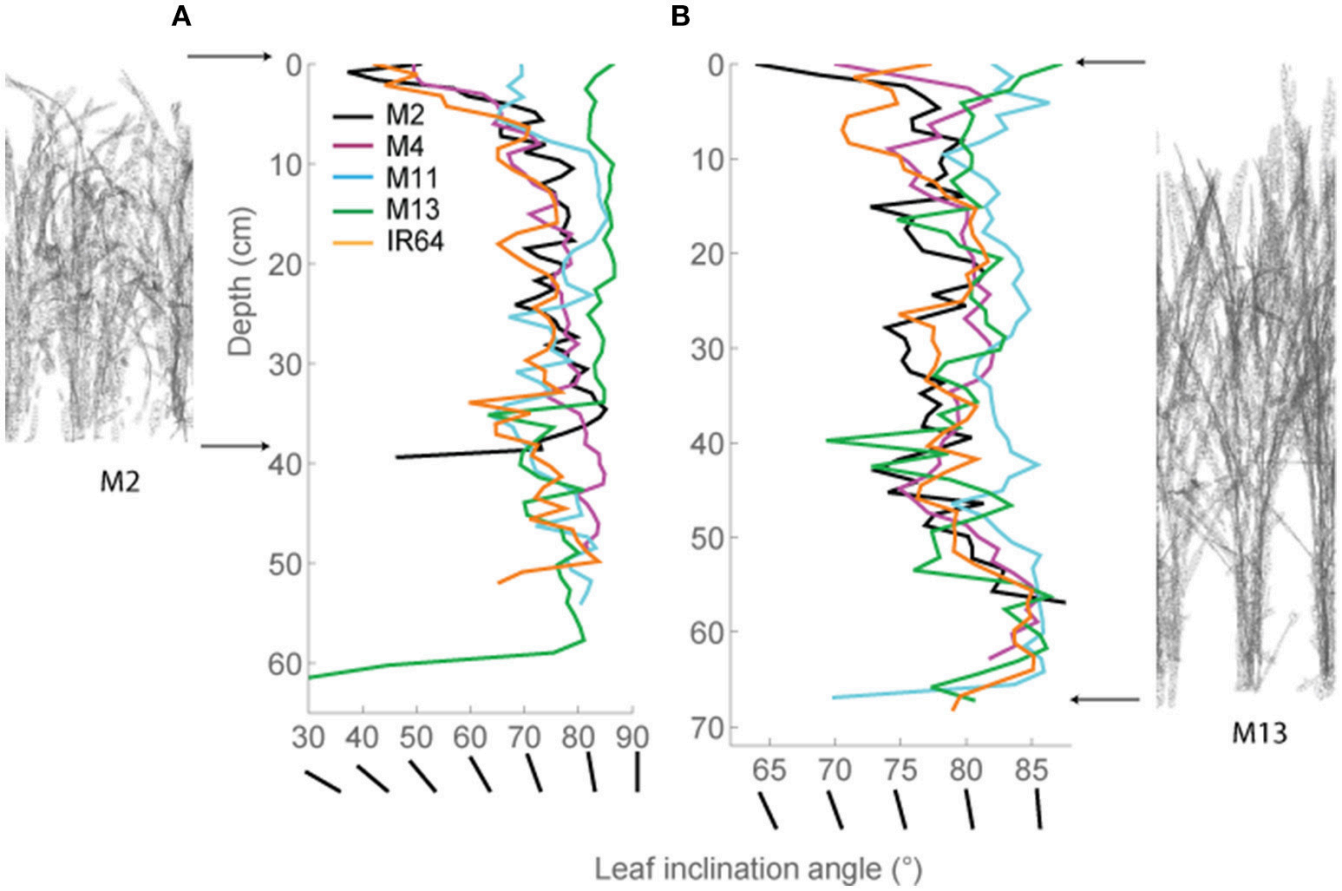
**Modeled leaf inclination angles throughout depth (i.e., distance from the top) in the canopy. (A)** GS1 and **(B)** GS2. Representations of M2 (left) and M13 (right) are shown at the side to make interpretation easier. As an additional visual aid, we have added lines of different angles which correspond to the leaf angles shown on the X-axis. The average triangle inclination angle throughout the horizontal subsection was calculated with respect to vertical, where a leaf inclination angle toward 0 indicates a more horizontal leaf and an inclination angle of 90 indicates a more vertical leaf. M2, M4, M11, and M13 refer to Shan-Huang Zhan-2 (SHZ-2), IR4630-22-2-5-1- 3, 157 WAB 56-125, and Inia Tacuari, respectively.

These distinctive patterns are partly as a result of architecture and arrangement, specifically angles of the leaves, within each canopy. This technique allows automatic and rapid calculation of leaf angle of every triangle in the reconstruction. Leaf angle distributions were calculated (Burgess et al., 2015) for each canopy and averaged at each canopy depth (see Section Modeling; Figures 3A,B), where a leaf inclination angle toward 0 indicates a more horizontal leaf and an inclination angle of 90 indicates a more vertical leaf. M2, M4, and IR64 lines exhibited a trend toward more horizontal leaves at base of canopy at both growth stages 1 and 2, with M11 and M13 more vertical stature.

### Light environment

#### Modeled data

**Figure 4:**
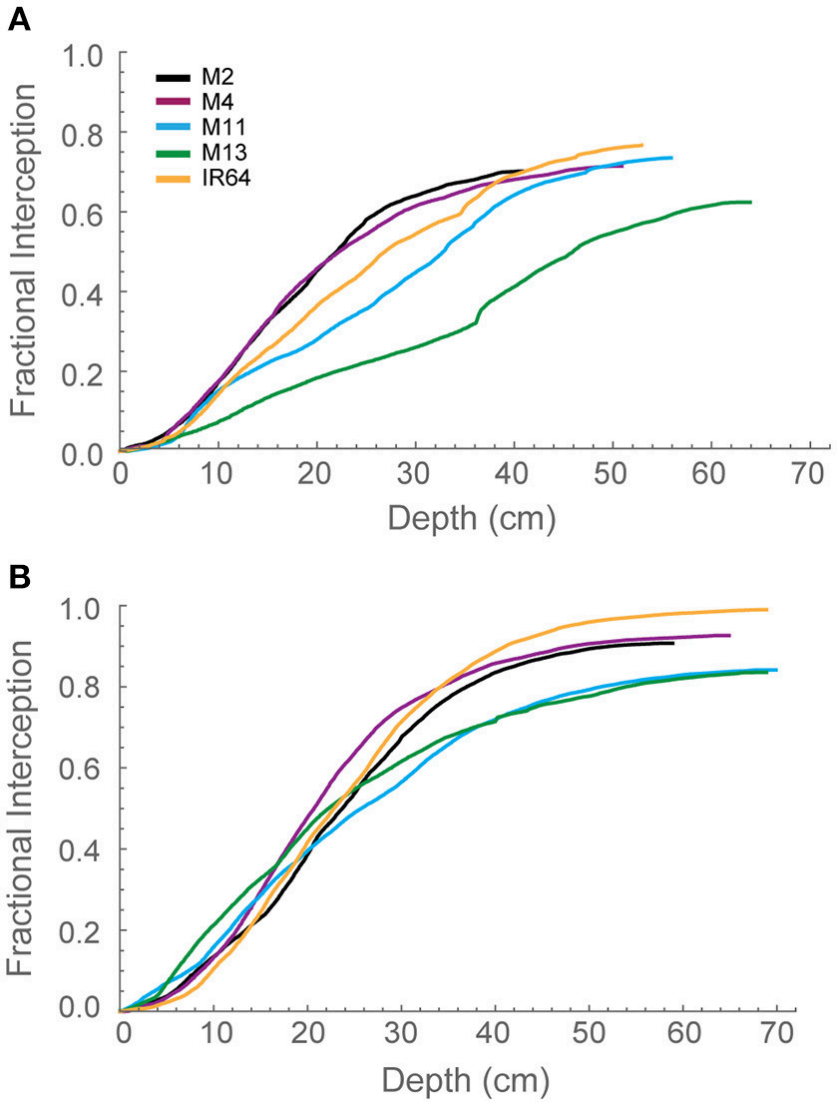
**Modeled fractional interception as a function of depth in the canopy at 12:00 h for (A)** GS1 and **(B)** GS2, using ray tracing data. Curves were calculated with step Δd = 1 mm. M2, M4, M11, and M13 refer to Shan-Huang Zhan-2 (SHZ-2), IR4630-22-2-5-1-3, 157 WAB 56-125, and Inia Tacuari, respectively.

To explore interactions between depth and light interception, modeled fractional interception (FI) was calculated as a function of depth (Figures 4A,B). This enables the interception to be calculated at a resolution of 1 mm throughout the canopy. Generally, the pattern was similar to that of modeled LAI. At GS1 (Figure 4A), M2, and M4 are achieving ~60% of interception within the top 25 cm of the canopy. This can be compared to M13, which exhibits a near linear relationship between FI and canopy depth. By GS2 (Figure 4B), the lines exhibit a more similar interception within the top 20 cm of the canopy but a greater variation in the bottom layers in the canopy. M2, M4, and IR64 achieve the greatest FI and M11 and M13 the lowest.

We hypothesize that leaf angle will be related to vertical FI and LAI distribution: we note that toward the top of the canopy, leaves tend to be more horizontal (i.e., angles approaching 0) for those lines with a higher LAI (Figures 2, 3), and this contributes to a higher interception of light (Figure 4). In the lines studied here, erectness does not seem to be associated with a higher LAI.

### Photosynthesis

#### Measured data

There were no significant differences between any of the ACi curve parameters (*V*_*cmax*_, J, and TPU) at either growth stage (see Table 2). There was a significant difference in Chlorophyll a content (*P* = 0.034) and total chlorophyll content (*P* = 0.041) between the lines with M11 and M13 containing the highest levels and Chl a:b ratios showing little change (Table 3). The dark-adapted Fv/Fm measurement measured at the top of the canopy also shows significant differences between the lines at both growth stages under two different weather conditions, full sun and cloudy with supplementary lights, (*P* < 0.002 for all) with the lowest Fv/Fm-value found in M2 (Table 4). This is in agreement with previous work on canopy architecture and susceptibility of plants to photoinhibition, whereby erect architectures are less susceptible to high light and have a higher Fv/Fm in accordance with Burgess et al. (2015). Lowered Fv/Fm are seen under high irradiance in healthy rice and wheat plants in the field and represent a decline in maximum photosystem II quantum yield, caused either by damage to reaction centers or another form of sustained quenching (Murchie et al., 1999; Burgess et al., 2015).

**Table 2:** **Parameters taken from ACi curve fitting at GS1 (45 DAT) using Sharkey et al. (2007) (fitting at 30°C)**.

**Line**	**Layer**	**V_cmax_**	**J**	**TPU**
M2	Top	140.5 ± 13.4	187.6 ± 11.1	13.1 ± 0.7
M4		145.9 ± 18.0	202.7 ± 9.0	13.7 ± 0.6
M11		135.6 ± 12.0	195.8 ± 16.3	12.9 ± 1.0
M13		143.4 ± 12.3	186.9 ± 12.3	12.4 ± 0.5
IR64		134.8 ± 12.7	181.3 ± 9.2	12.0 ± 0.6
Mean		140	190.9	12.82
*P*		0.982	0.847	0.695
SED		22.23	20.45	1.21
M2	Bottom	120.4 ± 8.0	173.1 ± 9.1	11.5 ± 0.8
M4		131.4 ± 19.9	180.2 ± 11.8	11.8 ± 0.5
M11		127.3 ± 10.8	201.6 ± 24.9	13.0 ± 0.8
M13		141.2 ± 17.0	182.0 ± 6.9	11.6 ± 0.5
IR64		126.1 ± 15.7	166.3 ± 11.0	11.4 ± 0.9
Mean		129.3	180.6	11.83
*P*		0.905	0.606	0.551
SED		22.05	22.07	1.05

*The means of six independent curves are shown with standard errors of the mean. P-value corresponds to ANOVA. M2, M4, M11, and M13 refer to Shan-Huang Zhan-2 (SHZ-2), IR4630-22-2-5-1-3, 157 WAB 56–125, and Inia Tacuari, respectively*.

**Table 3:** **Chlorophyll content and chlorophyll a:b ratio at GS2 (85 DAT), top of canopy**.

**Line**	**Chl a (μg/cm^2^)**	**Chl b (μg/cm^2^)**	**Chl a+b (μg/cm^2^)**	**Chl a:b**
M2	36.10 ± 2.40	8.46 ± 0.55	44.56 ± 2.92	4.27 ± 0.08
M4	36.53 ± 2.71	8.93 ± 0.85	45.46 ± 3.43	4.19 ± 0.19
M11	45.67 ± 3.78	10.30 ± 0.80	55.98 ± 4.57	4.42 ± 0.07
M13	53.69 ± 2.61	11.70 ± 0.50	65.40 ± 3.08	4.58 ± 0.08
IR64	39.01 ± 1.71	9.19 ± 0.39	48.20 ± 2.06	4.25 ± 0.09
Mean	42.2	9.72	51.9	4.344
*P*	0.034	0.126	0.041	0.356
SED	5.28	1.20	6.41	0.20

*The means of three plots are shown with standard errors of the mean. P-value corresponds to ANOVA. M2, M4, M11, and M13 refer to Shan-Huang Zhan-2 (SHZ-2), IR4630-22-2-5-1-3, 157 WAB 56–125, and Inia Tacuari, respectively*.

**Table 4:** **Maximum quantum yield of PSII (Fv/Fm) measured after 20 min dark adaptation**.

**Line**	**GS1**		**GS2**	
	**Full sun**	**Clouds + sup**	**Full sun**	**Clouds + sup**
		**lights**		**lights**
M2	0.748 ± 0.009	0.780 ± 0.010	0.788 ± 0.005	0.801 ± 0.004
M4	0.785 ± 0.004	0.805 ± 0.003	0.803 ± 0.007	0.830 ± 0.006
M11	0.813 ± 0.001	0.828 ± 0.004	0.810 ± 0.007	0.838 ± 0.006
M13	0.814 ± 0.013	0.848 ± 0.009	0.841 ± 0.007	0.846 ± 0.004
IR64	0.792 ± 0.007	0.816 ± 0.003	0.816 ± 0.003	0.826 ± 0.003
Mean	0.791	0.819	0.812	0.828
*P*	0.001	0.002	0.001	<0.001
SED	0.0115	0.0090	0.0084	0.0067

*Five measurements were taken per plot. The means of three plots are shown with standard errors of the mean. Growth stage 1 corresponds to 45 DAT and 2 at 85 DAT. M2, M4, M11, and M13 refer to Shan-Huang Zhan-2 (SHZ-2), IR4630-22-2-5-1-3, 157 WAB 56–125, and Inia Tacuari, respectively*.

**Figure 5:**
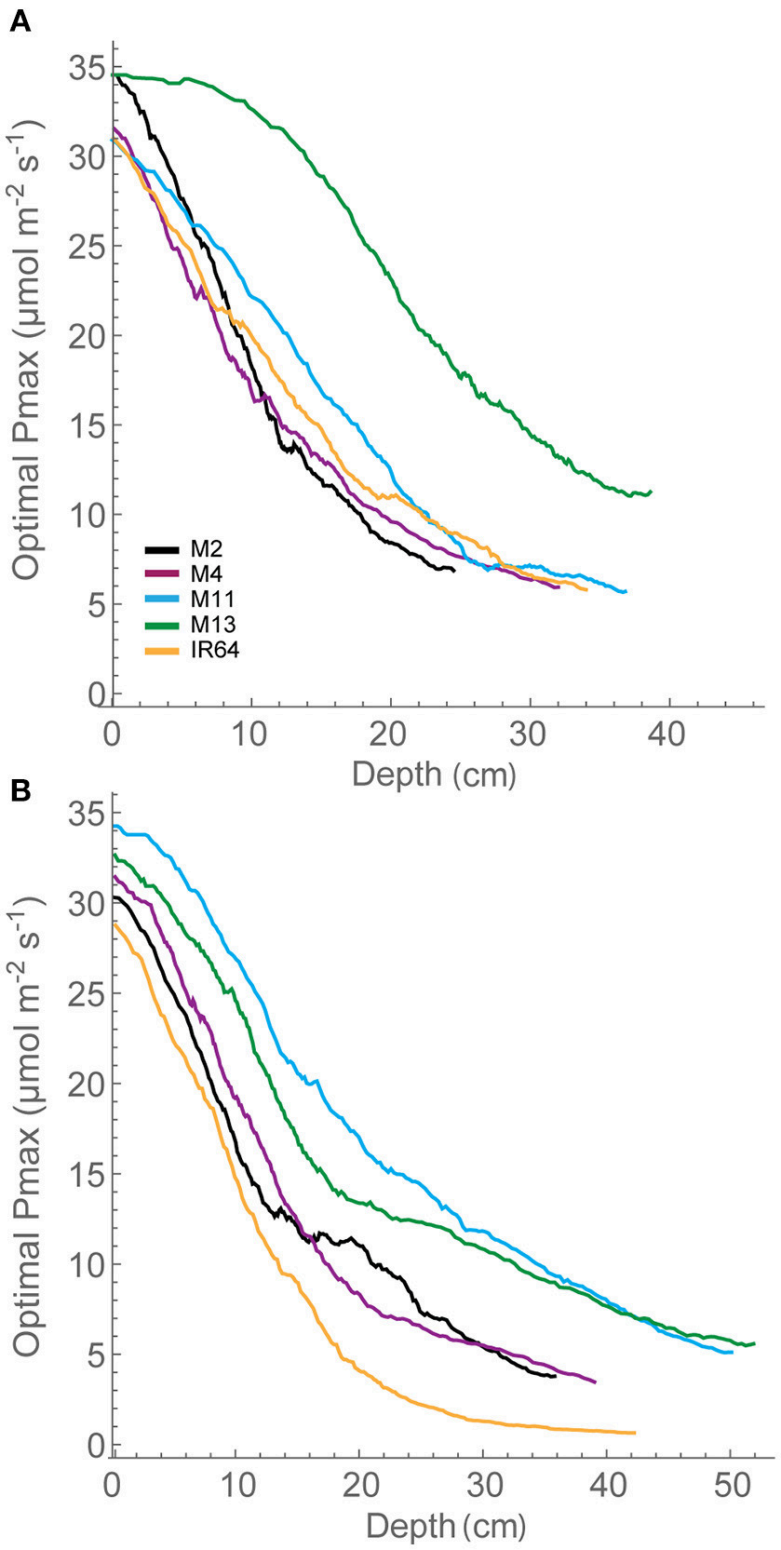
**Whole canopy acclimation model output**. The acclimation model was run at 250 locations throughout canopy depth to predict the optimal *P*_*max*_ at each location throughout canopy depth (i.e., from the top of the canopy) dependent upon the light environment that it experienced, calculated via ray tracing. A moving average has been fitted to the data. **(A)** GS1 and **(B)** GS2. M2, M4, M11, and M13 refer to Shan-Huang Zhan-2 (SHZ-2), IR4630-22-2-5-1-3, 157 WAB 56-125, and Inia Tacuari, respectively.

We assessed photosynthesis at different canopy layers and compared it to patterns of LAI accumulation above. *P*_*max*_ for the top layer varied between species for GS1 (*P* < 0.001), with M13 having a higher *P*_*max*_ than M4, but not GS2 (*P* = 0.053; Table 2). There was no significant difference in *P*_*max*_ for the bottom layer at either growth stage (*P* = 0.062 for GS1 and *P* = 0.321 for GS2). There were no apparent consistencies between canopy structure and distribution of *P*_*max*_ except that the highest *P*_*max*_, and the largest decline in *P*_*max*_ for the top layer between GS1 and 2 is shown by M13; the line with the lowest cumulative LAI (Figure 5).

#### Modeled data

An empirical model of photosynthesis was employed to calculate the total canopy carbon gain per unit leaf area and per unit ground area (see Section Materials and Methods); results are presented in Table 2. For GS1, M13 exhibits the highest carbon gain per unit leaf area followed by M2 and M4, respectively, with IR64 showing the lowest value. For carbon gain per unit ground area, M13 remains the highest, followed by M2 and M11. This can be attributed to the higher *P*_*max*_ for that line, despite the reduced LAI. At GS2, all canopies show a reduced carbon gain per unit leaf area and increased carbon gain per unit ground area. This is presumably due to an increase in LAI of all canopies and a concurrent increase in proportion of shaded leaves. Per unit leaf area M11 and M13 show the highest values of carbon gain and per unit ground area M11 is the highest, followed by M2 and M13. However, we saw only weak correlations between *P*_*max*_ and carbon gain per unit leaf area and ground area (Supplementary Figure S2).

Canopy structures result in dynamic fluctuations in light from solar movement. The different architectures studied here are likely to generate different characteristics of fluctuations, in addition to the light interception shown above (Burgess et al., 2015). The most appropriate approach is a functional analysis of this variation in dynamic light via the impact that it has on the predicted distribution of a modeled optimal *P*_*max*_. This was calculated using an empirical model of acclimation (see Section Modeling; Retkute et al., 2015). The model takes into account the fluctuating light over a full day within the canopy and provides an optimal *P*_*max*_; the value of *P*_*max*_ that is optimized in terms of carbon gain for that particular light pattern, if light were the sole determinant, using the frequency and duration of high light periods. This differs from previous models that use integrated light over the whole day (e.g., Stegemann et al., 1999). Thus, the optimal *P*_*max*_ provides a means of analyzing both the frequency and duration of high light events in the canopy.

The distribution in optimal *P*_*max*_ for each of the canopies is given in Figure 5. This shows distinctive differences between the lines. At GS1, M4, M11, and IR64 show similar patterns for distribution of optimal photosynthetic capacity. These rank in the same order as FI and LAI for depths of 15–35 cm, with lower FI and LAI leading to higher optimal *P*_*max*_, as one would expect. M13 with its upright leaves and more open canopy shows a similar pattern for reduction in optimal *P*_*max*_ throughout but a greater value achieved at all canopy layers (depths) and a plateau in optimal *P*_*max*_ toward the top of the canopy. By GS2, differences between each of the canopies are less obvious. All canopies exhibit similar steep gradients within the top section of the canopy followed by a shallower gradient at the bottom of the canopy. IR64 has the lowest predicted optimal *P*_*max*_-values of all canopies with the bottom ~40 cm under 5 μmol m^−2^ s^−1^. However, the ranking is still persistent, this time at lower canopy regions >40 cm. This indicates that optimal *P*_*max*_ can be consistently related to these features of canopy architecture. However, the relationship with leaf angle is less obvious. Measured *P*_*max*_-values in the lower regions of the canopy were higher than the predicted optimal *P*_*max*_.

## Discussion

### Canopy reconstructions

Plant canopies often consist of an assemblage of structurally diverse plants with particular spatial distributions of photosynthetic material. The way in which these photosynthetic surfaces intercept light energy and assimilate CO_2_ is the basis for whole canopy photosynthesis, and thus the arrangement of plant material that optimizes light interception will inherently lead to increased productivity. If all incident light is absorbed (FI = 1) then whole canopy photosynthesis is a result of the efficiency of distribution of light across a particular LAI. The architectures of five diverse rice cultivars at two different growth stages were captured using a low-tech method for high-resolution canopy reconstruction. This reconstruction method has previously been shown to provide an accurate representation of the plants with replication of leaf area between 1 and 4% of that of measured data and accurate capture of leaf angles (Pound et al., 2014; Burgess et al., 2015). In combination with ray tracing using fastTracer3, the reconstruction method provides an accurate depiction of the light gradients found within real life canopies in field settings (Burgess et al., 2015). The structural differences (i.e., cLAI and leaf angle distributions) between diverse rice lines and their relationship to whole canopy photosynthesis can be explored in more depth using this modeling approach than would be possible using manual methods under field conditions.

### The relationship between canopy architecture and photosynthesis

To investigate the relationships between architectural features and photosynthetic traits, a correlation matrix was produced for manually measured data. Significant correlations (both positive and negative, given in bold) relating to canopy architectural features are given in Table 5. Among the factors that influence photosynthesis [here associated with *P*_*max*_ for the top (T) and bottom (B) canopy layers] are: tiller number, plant height, leaf number, and leaf width. However, these relationships are only significant at the first growth stage, not the second, indicating (i) the architecture at certain developmental stages (smaller plants) are more critical in determining photosynthesis characteristics, (ii) beyond a certain developmental stage, or a certain amount of leaf area, the levels of light inside the canopy are below a certain threshold so as to not significantly influence photosynthetic characteristics in particular acclimation to light intensity or (iii) photosynthetic performance is determined by factors other than architectural traits. Given the data concerning optimal *P*_*max*_ it seems possible that all of these suggestions could be contributing, as we explain below.

**Table 5:** **The relationship between measured canopy architectural traits and photosynthesis: the sample correlation coefficient value taken from the correlation matrix output for select canopy architectural and physiological traits**.

**Growth Stage**		**Tiller number**	**Plant height**	**Leaf area**	**Leaf number**	**Leaf width (B)**	**Leaf Width (T)**
1	Plant Height	–0.638^*^	**–**				
	Leaf Area	0.412	**–**0.221	**–**			
	Leaf Number	0.890^*^	–0.629^*^	0.521^*^	**–**		
	Leaf Width (B)	**–**0.240	0.601^*^	**–**0.045	**–**0.420	**–**	
	Leaf Width (T)	–0.907^*^	0.635^*^	**–**0.358	–0.813^*^	0.445	**–**
	*P_*max*_* (B)	–0.574^*^	0.601^*^	0.112	**–**0.425	0.513	0.519^*^
	*P_*max*_* (T)	–0.721^*^	0.730^*^	**–**0.189	–0.624^*^	0.626^*^	0.737^*^
	Fv/Fm Sun	–0.561^*^	0.830^*^	0.066	–0.585^*^	0.480	0.555^*^
	Fv/Fm Cloudy	–0.755^*^	0.881^*^	**–**0.150	–0.692^*^	0.589^*^	0.675^*^
	FI/Height	0.101	–0.713^*^	**–**0.012	0.158	–0.578^*^	**–**0.2145
2	Leaf Area	0.389	**–**0.053	**–**			
	Leaf Number	0.689^*^	**–**0.166	0.663^*^	**–**		
	Leaf Width (B)	–0.615^*^	0.408	**–**0.307	–0.627^*^	**–**	
	Leaf Width (T)	–0.819^*^	0.673^*^	**–**0.413	–0.652^*^	0.683^*^	**–**
	*P_*max*_* (B)	–0.524^*^	0.587^*^	**–**0.357	**–**0.645	0.706^*^	0.825^*^
	*P_*max*_* (T)	**–**0.311	0.453	**–**0.088	0.165	**–**0.065	0.176
	Fv/Fm Sun	–0.736^*^	0.486	**–**0.040	**–**0.195	0.294	0.694^*^
	Fv/Fm Cloudy	–0.709^*^	0.661^*^	**–**0.236	**–**0.382	0.441	0.734^*^
	Chl a	–0.752^*^	0.5645^*^	**–**0.251	**–**0.470	0.401	0.689^*^
	Chl b	–0.692^*^	0.619^*^	**–**0.290	**–**0.453	0.329	0.643^*^
	Total Chl	–0.7467^*^	0.5772^*^	**–**0.2587	**–**0.4702	0.3913	0.6855^*^
	FI/height	**–**0.2241	–0.5415^*^	**–**0.1975	**–**0.3912	0.2445	**–**0.0507

*Growth stage 1 corresponds to 45 DAT and 2 at 85 DAT. (T) corresponds to measurements from the top canopy layer and (B) from the bottom canopy layer. FI/Height refers to fractional interception as a function of height throughout the canopy. Significant correlations are given in bold*,

* *indicates P < 0.05*.

*Correlations based on plot means. Dry weight was not significantly correlated to any trait and so is not shown*.

There is a positive correlation, although weak, between plant height and photosynthesis during GS1, which may be initially contrary to what would be expected. Whilst extra height may provide an advantage during competition with shorter neighbors (such as weeds in Upland cultivars), it is also possible that height may increase self-shading over a greater surface area of the canopy, thus could intuitively reduce canopy productivity (diffuse light notwithstanding). Alternatively, plant height could be linked closely with leaf angles, with taller plants containing more elongated and erect leaves (as seen within our two tallest study lines: M11 and M13), which can lead to greater penetration of light throughout the canopy especially at mid-day, despite the greater height. Conversely, increased photosynthetic potential could provide plants with the means to achieve greater height. There is increasing evidence that tall plants provide greater sinks for photosynthate (i.e., within the stems) that can reduce limitations based on source-sink processes. This can lead to higher photosynthetic rates, at the leaf level, within taller crops. Therefore, the positive correlation between plant height and photosynthesis at GS1 could be a result of stem sink development during this stage.

To explore how canopy architecture influences photosynthesis and light interception at the whole canopy level, a line of best fit between measured LAI and modeled data were made (Supplementary Figure S2). Total canopy light interception is negatively correlated to measured LAI at both growth stages (*R*^2^ = 0.981 and 0.967 for GS1 and GS2, respectively). Similarly, there is also a negative correlation between measured LAI and carbon gain per unit leaf area (*R*^2^ = 0.775 and 0.914 for GS1 and GS2, respectively). Thus across the five rice lines, an increase in leaf area leads to a decrease in total light intercepted and in carbon gain per unit leaf area, possibly representing the “dilution effect” (Field and Mooney, 1983), although this does not translate to a significant decrease in measured *P*_*max*_ (Table 6), nor does it translate into an effect on carbon gain per unit ground area, with no clear relationship at either growth stage (*R*^2^ = 0.311 and 0.091 for GS1 and GS2, respectively).

**Table 6:** **Gas exchange and modeling results at each growth stage**.

**Growth stage**	**Line**	***P_*max*_* top layer (μmol m^−2^ s^−1^)**	***P_*max*_* bottom layer (μmol m^−2^ s^−1^)**	**Carbon gain per unit leaf area (mol m^−2^ d^−1^)**	**Carbon gain per unit ground area (mol m^−2^ d^−1^)**	**Total light interception (mol m^−2^ d^−1^)**
1	M2	24.29 ± 1.61	20.23 ± 1.69	0.241	0.532	11.98
	M4	22.67 ± 1.76	17.91 ± 1.82	0.220	0.489	12.25
	M11	29.99 ± 2.37	24.52 ± 2.53	0.204	0.504	11.16
	M13	38.65 ± 2.82	27.34 ± 3.54	0.432	0.798	13.34
	IR64	25.96 ± 1.63	20.24 ± 1.70	0.169	0.480	10.18
	Mean	28.31	22.1			
	*P*	<0.001	0.062			
	SED	2.96	3.34			
2	M2	20.15 ± 0.77	14.14 ± 1.82	0.174	0.827	7.08
	M4	22.67 ± 1.78	14.78 ± 1.87	0.121	0.661	6.28
	M11	26.83 ± 2.72	17.69 ± 1.63	0.232	0.968	7.72
	M13	23.53 ± 1.11	18.83 ± 1.34	0.236	0.828	8.57
	IR64	26.35 ± 1.02	15.90 ± 1715	0.082	0.714	4.08
	Mean	23.91	16.33			
	*P*	0.053	0.321			
	SED	2.32	2.39			

*Measured P_max_ for the top and bottom layer was calculated from light response curve fitting; the means of six (GS1) or five (GS2) measurements are shown with standard errors of the mean. P-value corresponds to ANOVA. An empirical model of photosynthesis was employed to calculate carbon gain per unit leaf area and ground area using light levels predicted by ray tracing for 10th December (GS1) and 21st January (GS2), respectively (see Section Imaging and Ray Tracing). Total light interception over the course of the day was also calculated. Growth stage 1 corresponds to 45 DAT and 2 at 85 DAT. M2, M4, M11, and M13 refer to Shan-Huang Zhan-2 (SHZ-2), IR4630-22-2-5-1-3, 157 WAB 56–125, and Inia Tacuari, respectively*.

This lack of a relationship may be due to a high canopy density, high nutrient accumulation within the canopy leading to a large proportion of shaded leaves with a high respiratory burden (see below; Reich et al., 1998). It might be expected that leaf angle, canopy light interception and LAI distribution are closely related: indeed this was shown in Figures 2–4 at depths between 10 and 30 cm (e.g., where M11 and M13 have lowest LAI and F but highest leaf angle). The conclusion is that a more upright leaf angle permits a greater light penetration but a greater LAI accumulation at GS2 lessens this effect. This is consistent with previous work (e.g., Song et al., 2013).

The dynamic light pattern cast by canopies presents a complex problem: how do leaves determine the optimal properties of a light response curve for a given time period? We used a model that predicts the optimal *P*_*max*_ based on ray tracing throughout the canopy depth. The optimal *P*_*max*_ distribution (Figure 5) follows a similar pattern (in terms of ranking responses among lines) to LAI and FI at the first growth stage. The ranking similarity is not so clear in the second, see above comment regarding *P*_*max*_ measurements. The differences between each of the lines, particularly at the first growth stage, indicate that whilst the quantity of leaf material (i.e., the LAI) may be similar, the arrangement of this material in 3-dimensional space can lead to dramatic changes in carbon assimilation in different canopy layers.

The greater potential optimal *P*_*max*_ at the bottom of the canopy in M13 at GS1 relative to the other varieties can be linked to the low accumulation of leaf material with canopy depth (as seen with cLAI; Figures 2A,B) and the reduced FI of light (Figure 4) but an increased total light intercepted over the whole canopy (Table 6). This suggests that architecture which enables greater light penetration to lower canopy layers leads to a greater assimilation of carbon at lower canopy layers, which contributes to overall canopy photosynthesis. This is seen as an increased carbon gain per unit leaf area relative to the other lines (Table 6). However, when assessing the carbon assimilation per unit ground area, M13 ranks in the middle of the five varieties, indicating that despite the open canopy and greater light penetration, the reduced LAI of the variety leads to reduced productivity on a per land area basis. This indicates a small level of consistency between diverse canopy architectural traits and the long-term responses of photosynthesis to the light environment in this study. It shows that the architectural traits measured and modeled in this study are having a consistent impact on the light dynamics within the canopy, albeit over a limited number of genotypes. However, it is not possible to conclude whether it is possible to predict acclimation state from the distribution of FI and LAI within the canopy without detailed direct photosynthetic analysis of a wider range of genotypes.

When predicting optimal *P*_*max*_ we assumed that light dynamics are the sole factor determining photosynthetic capacity and that canopy nitrogen profiles correlate with canopy photosynthesis profiles. However, nitrogen profiles are frequently suboptimal with respect to photosynthesis (Hikosaka, 2016). The optimal *P*_*max*_ measurement is therefore a novel and potentially useful method for indicating photosynthetic nitrogen use efficiency in crop canopies, clearly shown here for all lines, even M13 with its more efficient light penetration. It needs to be pointed out that the use of the “time weighted average” or τ that was fixed at 0.2 was chosen to represent the time taken for photosynthetic induction, but we do not know whether acclimation status according to canopy position will have an effect on this.

The leaf inclination angle is critical in determining the flux of solar radiation per unit leaf area (Ehleringer and Werk, 1986; Ezcurra et al., 1991; Falster and Westoby, 2003). Plants containing steep leaf inclination angles tend to have a decreased light capture when the sun is directly overhead (i.e., during midday hours or during summer) but increases light capture at lower solar angles (i.e., start/end of the day or during seasonal changes in the higher latitude regions). This feature has a number of practical applications including the decrease in susceptibility to photoinhibition (Ryel et al., 1993; Valladares and Pugnaire, 1999; Werner et al., 2001; Burgess et al., 2015); reduced risk of overheating due to reduction in mid-day heat loads (King, 1997); and minimized water-use relative to carbon gain (Cowan et al., 1982). This architecture feature, combined with a relatively open canopy, has been adopted within our studied line; M13, and contributes to its inherent heat tolerance and higher Fv/Fm-values (Figure 3, Table 4). The erect leaf stature and higher Fv/Fm is also present in our studied line M11 (Figure 3, Table 4). This may suggest a relationship between erectness, maximum quantum yield, and latitude of origin of the lines with M11 and M13 originating in locations closer to the equator [Latin America including equatorial regions and WARDA (now AfricaRice), Western Africa, respectively] relative to the other lines. Such characteristics are in line with previous work to predict the optimal leaf angle according to latitude (Herbert, 2003; Baldocchi, 2005) and work in *Arabidopsis thaliana* (Hopkins et al., 2008). Correlations between architectural traits and latitude have also been seen within tree species, with a linear decrease in petiole length with an increase in latitude and change in leaf arrangement (King and Maindonald, 1999). The differences in Fv/Fm between the varieties may also be linked to the genetic background of the lines M11 and M13 with the *japonica* background and M2, M4 and IR64 with the *indica* background. This is in agreement with previous work on rice with higher Fv/Fm-values found in *japonica* cultivars relative to *indica* (Kasajima et al., 2011). Differences in Fv/Fm between the two groups are also mirrored in the capacity for non-photochemical quenching (NPQ) for energy dissipation, with much higher NPQ-values found in *japonica* lines (Kasajima et al., 2011).

Rice cultivation areas are highly diverse and are affected in differing ways by fluctuations in environmental conditions. Thus, the origin of each of the parental founders may also indicate why these specific architectural traits are present and how they interact with leaf photosynthetic properties. The five lines selected for this study have diverse origins including China (M2), South East Asia (International Rice Research Institute; M4 and IR64), Africa (M13), and Latin America (M11). The rapid maturation and early flowering of M13 relates to the short-growing seasons of upland rice production in Western Africa whilst stable yields under low nitrogen inputs enables relatively high yields under low-input upland systems (Gridley et al., 2002). Whilst there is little data relating to canopy architecture in divergent rice lines grown across the world, there has been some work done studying architectural differences between key African and Australian savannah tree taxa (Moncrieff et al., 2014). They found distinct differences between the two sets of taxa in key architectural traits including plant height and canopy area, and attributed the differences not to disparities in the environmental conditions in which the trees grew, but rather in the differing evolutionary history of African vs. Australian savannas. This may indicate that when assessing regional differences in rice architecture, we must take into account not only the biotic and abiotic differences between areas but also the biogeography, interactions with other species and historic cultivation practices.

Structure function relationships in terms of canopy architecture are complex and affected by growing environment. Many factors, in addition to the ideotype principle, will shape the commercial breeder's decision making process. There may be negative linkages with a particular trait (Rasmusson, 1991). Erect leaved ideotypes do not necessarily perform (Breseghello and Coelho, 2013) and architecture “performance” depends on location and environmental factors, inputs, and agronomy (Hammer et al., 2009). The erect ideotype means that a higher LAI and hence higher canopy photosynthesis could be supported but this also requires a high fertilizer (especially nitrogen) input which raises cost and reduces sustainability.

This is the first high-resolution study that has been used to attempt to assess the link between canopy architecture and photosynthesis characteristics. One of the drawbacks of this study was the inability to grow the lines in the location they originated, or under a range of different environments. This poses a challenge as canopy architecture is determined by a combination of the genetics of plant but also the conditions in which the plant was grown, including climate, weather patterns, soil type and the competitive presence of neighboring plants. Thus, the architecture adopted by the genotypes in this study may not be totally representative to that when grown elsewhere due to differences in growing conditions. In this study, we used the latitude of the Philippines as a fastTracer3 parameter as a standard to compare the different lines, which will be a different light environment to those in which the plants were grown or in which the lines traditionally grow or have originated. However, the conditions we used were enough to expose significant differences in architecture between lines which are genetically different in origin.

Other factors relating to whole canopy photosynthesis must also be taken into account such as: the angular relationship between the photosynthetic leaf surfaces and the sun; environmental conditions (i.e., wind speed, temperature, CO_2_ concentration); soil properties; the photosynthetic pathway used and; the presence of other biotic or abiotic stressors (Baldocchi and Amthor, 2001). This highlights the need for more in depth studies of canopy photosynthesis and architecture within the range of different environmental conditions in which a plant is likely to be exposed to. Also for more realistic modeling; i.e., modeling mimicking the weather conditions or more realistic representations of the plant stands in general (such as incorporating canopy movement due to wind: Burgess et al., 2016). These high-resolution studies will be critical in determining the exact relationships between canopy architectural features, photosynthesis, the light environment and productivity of our cropping systems and will provide the framework necessary for any future improvements.

Use of the parental founders of an elite MAGIC population of rice leads to possibilities for future studies using a wider number of crosses and their progeny into the genetic control of specific architectural features or breeding attempts to produce an “optimal plant type.” Whilst the genetic control of certain architectural traits is relatively understood (e.g., Wang and Li, 2006; Busov et al., 2008; Neeraja et al., 2009; Pearce et al., 2011), the interactions between genotype, phenotype, management, and the environment are less well-known. These relationships are confounded further by the variability in weather patterns and the relatively unknown effects of climate change on our agricultural systems. However, combining high-resolution studies and crop simulations with new breeding methods and genetic modeling provides a promising future for accelerating the discovery and creation of new idealized plant types. Multi-parent populations provide an attractive background for study when combined with high-throughput SNP genotyping (Bandillo et al., 2013).

## Author contributions

TH performed the initial screening experiment with the assistance of AB; AB performed the second in depth study; AB performed all imaging and reconstruction plus most of the modeling work with the assistance of RR; AB and EM wrote the article with the contributions of the other authors.

## Funding

AB is supported by the CFF-UNMC Doctoral Training Programme (CFF-UNMC DTP) under BiomassPLUS Programme BioP1-006 and the University of Nottingham, School of Biosciences. This work was also supported by the Biotechnology and Biological Sciences Research Council (grant number BB/JOO3999/1).

### Conflict of interest statement

The authors declare that the research was conducted in the absence of any commercial or financial relationships that could be construed as a potential conflict of interest.

## Supplementary material

Supplementary Figure S1 **Example of a time-weighted light pattern at τ = 0.2 (black line) relative to a non-weighted line (i.e., τ = 0)**. The time weighted average (Equation 9) is an exponentially decaying weight used to represent the fact that photosynthesis is not able to respond instantaneously to a change in irradiance levels. If τ = 0 then a plant will able to instantaneously respond to a change in irradiance, whereas if τ > 0 the time-weighted average light pattern will relax over the timescale τ. Within this study, τ was fixed at 0.2.

Supplementary Figure S2 **Correlations for different parameters for the two growth stages**.

Supplementary Table S1 **Agronomic details on the 16 Parental Lines used to develop the *indica* and *japonica* MAGIC Populations**. Data for MAGIC lines taken from Bandillo et al. (2013). The four MAGIC lines plus IR64 selected for in depth study are given in bold.

Supplementary Table S2 **Physiological characteristics of the 15 parental MAGIC lines + IR64 used in the initial screening**. All measurements, apart from harvest dry weight and seed dry weight, were taken 55–60 days after transplanting (DAT), corresponding to the vegetative growth stage. SPAD and leaf discs for chlorophyll samples were taken on the last full expanded leaf. The means of three plots are shown with standard errors of the mean. The bold lines are those selected for use in the in depth study due to their contrasting physiological features.
