# Supplementary figures and images for: Exploring Relationships between Canopy Architecture, Light Distribution, and Photosynthesis in Contrasting Rice Genotypes Using 3D Canopy Reconstruction
Source: Front Plant Sci. 2017 May 17;8:734. doi: 10.3389/fpls.2017.00734 (PMC5434157; doi:10.3389/fpls.2017.00734)

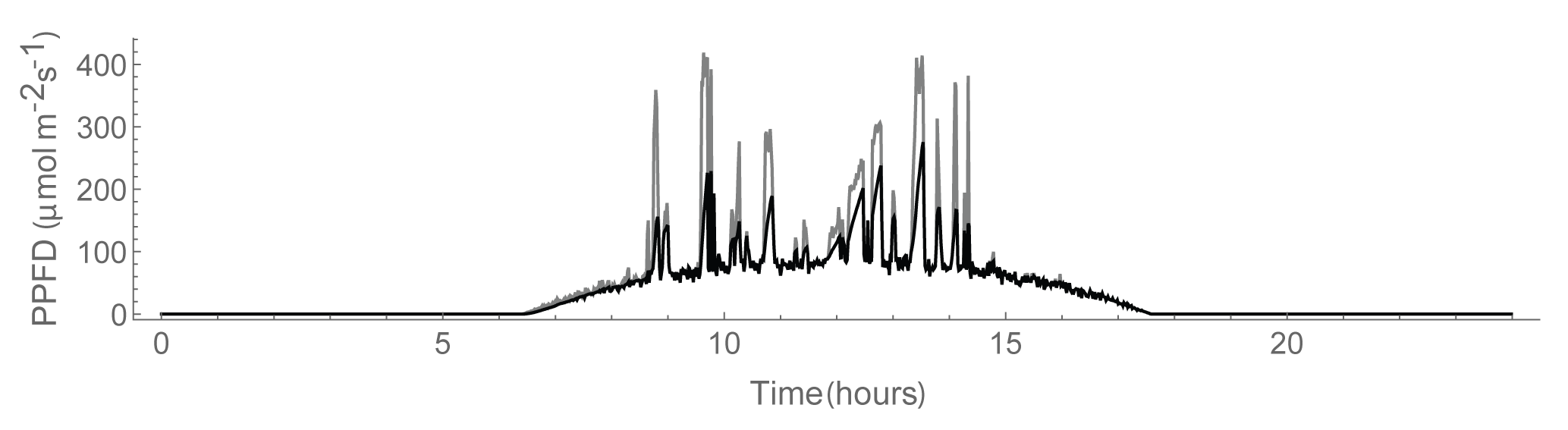

Supplement: Supplementary Figure S1 — Example of a time-weighted light pattern at τ = 0.2 (black line) relative to a non-weighted line (i.e., τ = 0). The time weighted average (Equation 9) is an exponentially decaying weight used to represent the fact that photosynthesis is not able to respond instantaneously to a change in irradiance levels. If τ = 0 then a plant will able to instantaneously respond to a change in irradiance, whereas if τ > 0 the time-weighted average light pattern will relax over the timescale τ. Within this study, τ was fixed at 0.2. [file Image1.TIF]

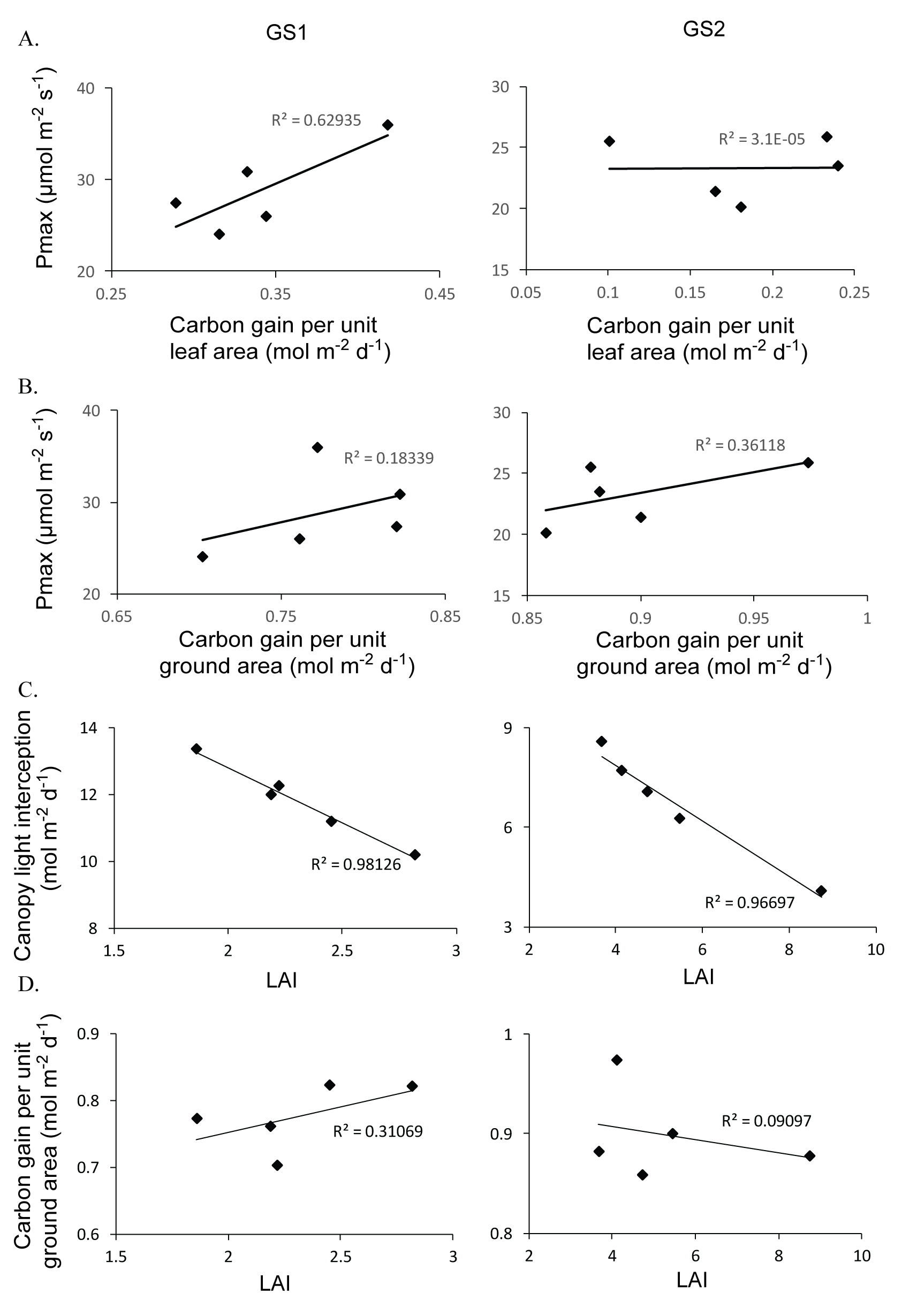

Supplement: Supplementary Figure S2 — Correlations for different parameters for the two growth stages. [file Image2.TIF]
