# Supplementary material for: Exploring Relationships between Canopy Architecture, Light Distribution, and Photosynthesis in Contrasting Rice Genotypes Using 3D Canopy Reconstruction
Source: Front Plant Sci. 2017 May 17;8:734. doi: 10.3389/fpls.2017.00734 (PMC5434157; doi:10.3389/fpls.2017.00734)
Supplement: Supplementary Table S1 — Agronomic details on the 16 Parental Lines used to develop the indica and japonica MAGIC Populations. Data for MAGIC lines taken from Bandillo et al. (2013). The four MAGIC lines plus IR64 selected for in depth study are given in bold. [file Table1.docx]

# Supplementary Tables

## Supplementary Table S1: Agronomic details on the 16 Parental Lines used to develop the *indica* and *japonica* MAGIC Populations.

| **ID** | **Name** | **Varietal Type** | **Origin** | **Relevance** |
| --- | --- | --- | --- | --- |
| **M1** | Fedearroz 50 | *indica* | Columbia | Popular and widespread. Stay green/ delayed senescence, disease tolerance, progenitor to many breeding lines |
| **M2** | Shan-Huang Zhan-2 (SHZ-2) | *indica* | China | Blast resistant, high yielding; in the pedigrees of many varieties in south China |
| **M3** | IR64633-87-2-2-3-3 (PSBRc82) | *indica* | IRRI | High yielding and most popular variety of the Philippines |
| **M4** | IR77186-122-2-2-3 (PSBRc 158) | *indica /* Tropical *japonica* | IRRI | High yielding variety in New Plant Type II background |
| **M5** | IR77298-14-1-2-10 | *indica* | IRRI | Drought tolerant in lowlands with IR64 background and tungro resistance |
| **M6** | IR4630-22-2-5-1-3 | *indica* | IRRI | Good plant type, salt tolerant at seedling and reproductive stages |
| **M7** | IR45427-2B-2-2B-1-1 | *indica* | IRRI | Fe toxicity tolerant |
| **M8** | Sambha Mahsuri + Sub1 | *indica* | IRRI | Mega variety with wide compatibility, good grain quality and submergence tolerance |
| **M9** | CSR 30 | Basmati group | India | Sodicity tolerance, Basmati type long aromatic grain |
| **M10** | Cypress | Tropical *japonica* | USA | High yielding, good grain quality and cold tolerant |
| **M11** | IAC 165 | Tropical *japonica* | Latin America | Aerobic rice adaptation |
| **M12** | Jinbubyeo | Temperate *japonica* | Korea | High yielding and cold tolerant |
| **M13** | WAB 56-125 | *O. glaberrima* in *indica* background | WARDA | NERICA background (*O. glaberrima*); heat tolerant and early flowering |
| **M14** | IR73571-3B-11-3-K2 | Tropical *japonica* x *indica* | IRRI-Korea Project | Tongil type, salinity tolerant |
| **M15** | Inia Tacuari | Tropical *japonica* | Uruguay | Earliness, wide adaptation and good grain quality |
| **M16** | Colombia XXI | Tropical *japonica* | Colombia | High yielding and delayed senescence |
| **IR64** | IR64 | *indica* | IRRI | High yielding, high grain quality, wide adaptability, disease resistance |

Data for MAGIC lines taken from (Bandillo *et al.,* 2013). The four MAGIC lines plus IR64 selected for in depth study are given in bold.

## Supplementary Table S2: Physiological characteristics of the 15 parental MAGIC lines + IR64 used in the initial screening.

| **Line** | **SPAD** | **Chlorophyll a:b** | **Chlorophyll content (a+b: μg cm^2^)** | **Plant Height (cm)** | **LAI**  **(m^2^ m^-2^)** | **Fresh Weight**  **(g plant^-1^)** | **Dry Weight**  **(g plant^-1^)** | **Harvest Dry Weight**  **(g plant^-1^)** | **Seed Dry Weight**  **(g plant^-1^)** |
| --- | --- | --- | --- | --- | --- | --- | --- | --- | --- |
| **M1** | 43.4±1.6 | 4.3±0.2 | 8.3±0.8 | 79.5±1.7 | 11.7±2.2 | 48.8±11.6 | 11.2±3.0 | 32.8±7.0 | 26.4±7.7 |
| **M2** | **38.4±0.8** | **3.6±0.1** | **7.4±0.1** | **71.8±1.3** | **7.7±2.2** | **38.5±10.2** | **8.8±2.1** | **37.9±6.3** | **22.2±5.0** |
| **M3** | 41.6±1.0 | 4.2±0.1 | 10.0±0.9 | 88.9±2.6 | 8.9±3.5 | 37.8±13.1 | 9.3±3.1 | 34.0±6.3 | 26.5±5.1 |
| **M4** | **41.4±0.8** | **3.9±0.1** | **10.7±1.2** | **74.4±2.5** | **6.0±1.3** | **28.2±6.4** | **6.4±1.4** | **28.2±6.4** | **8.0±3.1** |
| **M5** | 41.0±0.8 | 3.8±0.1 | 9.4±0.5 | 73.0±1.5 | 7.2±1.6 | 31.2±6.9 | 6.8±1.5 | 10.2±1.0 | 15.1±1.7 |
| **M6** | 39.5±1.4 | 4.1±0.0 | 9.5±0.6 | 71.4±2.9 | 7.3±2.1 | 33.8±8.9 | 7.9±1.9 | 22.8±3.2 | 14.6±2.3 |
| **M7** | 44.2±2.1 | 4.1±1.0 | 7.1±1.4 | 83.5±1.9 | 7.4±3.1 | 35.8±15.7 | 9.1±3.7 | 30.9±6.5 |  |
| **M8** | 42.1±1.8 | 4.0±0.2 | 7.4±1.0 | 85.5±1.3 | 8.1±1.7 | 40.1±7.4 | 9.1±1.7 | 18.0±2.6 | 7.9±0.7 |
| **M9** | 39.5±0.6 | 4.4±0.1 | 9.0±0.4 | 105.4±3.8 | 8.5±3.2 | 47.8±15.6 | 11.2±3.7 | 34.3±4.1 | 23.7±3.1 |
| **M10** | 48.2±0.6 | 3.8±0.4 | 7.2±0.7 | 95.5±1.6 | 5.4±2.3 | 30.1±9.2 | 8.2±2.6 |  |  |
| **M11** | **41.8±1.7** | **3.9±0.4** | **7.1±1.9** | **91.8±1.9** | **3.6±0.7** | **23.9±4.9** | **4.9±0.9** | **26.3±3.5** | **15.0±2.8** |
| **M12** | 39.1±1.7 | 4.1±0.1 | 7.9±1.0 | 78.5±2.2 | 5.7±0.3 | 26.1±0.4 | 6.4±0.3 | 20.5±2.0 | 14.1±1.1 |
| **M13** | **47.3±0.9** | **4.1±0.1** | **11.6±1.2** | **90.6±3.0** | **4.7±0.4** | **37.0±0.4** | **7.3±0.2** | **22.6±3.2** | **18.6±2.4** |
| **M14** | 41.5±2.4 | 3.8±0.1 | 7.8±0.9 | 87.5±2.1 | 4.1±0.2 | 26.7±2.6 | 5.7±0.7 | 15.8±2.6 | 16.1±3.7 |
| **M15** | 39.3±1.8 | 4.4±0.3 | 7.8±0.9 | 77.3±2.2 | 8.1±0.0 | 38.5±3.1 | 8.1±0.4 | 15.8±3.6 | 5.0±1.2 |
| **IR64** | **42.4±0.8** | **4.0±0.1** | **9.7±1.1** | **81.2±1.7** | **12.3±0.0** | **47.5±5.0** | **10.6±1.2** | **19.4±1.9** | **14.4±1.4** |

All measurements, apart from harvest dry weight and seed dry weight, were taken 55-60 days after transplanting (DAT), corresponding to the vegetative growth stage. SPAD and leaf discs for chlorophyll samples were taken on the last full expanded leaf. The means of three plots are shown with standard errors of the mean. The bold lines are those selected for use in the in depth study due to their contrasting physiological features
